# Natural soils in OECD 222 testing — influence of soil water and soil properties on earthworm reproduction toxicity of carbendazim

**DOI:** 10.1007/s10646-023-02636-9

**Published:** 2023-03-01

**Authors:** Eva Aderjan, Eiko Wagenhoff, Ellen Kandeler, Thomas Moser

**Affiliations:** 1 Eurofins Agroscience Services Ecotox GmbH, Eutinger Straße 24, 75223 Niefern-Öschelbronn, Germany; 2 University of Hohenheim, Institute of Soil Science and Land Evaluation, Emil-Wolff-Str. 27, 70599 Stuttgart, Germany

**Keywords:** Natural soil, Soil water, Earthworm, Toxicity, Carbendazim, Environmental risk assessment

## Abstract

Soil sorption properties can influence the bioavailability of substances and consequently the toxicity for soil organisms. Current standardised laboratory testing for the exposure assessment of pesticides to soil organisms uses OECD artificial soil that does not reflect the high variation in chemical-physical soil properties found in natural agroecosystems. According to guideline OECD 222, earthworm reproduction tests with *Eisenia fetida* and the pesticide carbendazim were performed in four natural soils and OECD artificial soil. By using pF 1.6, which ensures a uniformity in *actual soil water availability*, the control reproduction performance of *E. fetida* in all natural soils was at the same level as OECD artificial soil. In a principle component analysis, the variation in toxicity between the tested soils was attributable to a combination of two soil properties, namely total organic carbon content (TOC) and pH. The largest difference of 4.9-fold was found between the typical agricultural Luvisol with 1.03% TOC and pH 6.2 (EC_10_: 0.17 (0.12–0.21) mg a.i. kg^−1^ sdw, EC_50_: 0.36 (0.31–0.40) mg a.i. kg^−1^ sdw) and OECD artificial soil with 4.11% TOC and pH 5.6 (EC_10_: 0.84 (0.72–0.92) mg a.i. kg^−1^ sdw, EC_50_: 1.07 (0.99–1.15) mg a.i. kg^−1^ sdw). The use of typical agricultural soils in standardised laboratory earthworm testing was successfully established with using the measure pF for soil moisture adjustment. It provides a more application-oriented approach and could serve as a new tool to refine the environmental risk assessment at lower tier testing or in an intermediate tier based approach.

## Introduction

According to the current guidelines for environmental risk assessment (ERA) of plant protection products (PPP) for non-target organisms, standardised laboratory tests are carried out with the aim that the intended use of the product is safe (Schäfer et al. 2019). One of the most commonly used ecotoxicological tests for the soil compartment is the laboratory earthworm reproduction test with the model organisms *Eisenia fetida* and *Eisenia andrei* in artificial soil according to OECD 222 (2016) (EFSA 2017).

By using OECD artificial soil consisting of sand, kaolin, and peat, criteria for standardisation such as reproducibility and comparability are addressed. However, this artificial substrate cannot represent the diversity of natural soils, nor is it representative of arable soils to which PPP are usually applied. In 2017, the European Food Safety Authority (EFSA) issued a scientific opinion addressing the state of the science on risk assessment of PPP for in-soil organisms, stating that a standardised arable soil closer to the scenarios in the exposure assessment would be preferred over the artificial soil.

In the current tier-based approach of the ERA, the toxicity exposure ratio (TER) is calculated from laboratory results and the predicted environmental concentration (PEC). If the TER falls below a fixed safety factor, then higher-tier studies must be carried out as further testing requirements in the approval procedure of PPP (Schäfer et al. 2019). In these earthworm field studies performed according to ISO 11268-3 (2015) guideline, it is recommended to avoid using extreme soil types like “very sandy, clay, or moory soils”. Further criteria for the soil properties are not set in this guideline.

Soil properties such as texture and soil organic matter content have a major impact on the sorption and fate of substances in soils. The sorption properties of a soil are influencing the bioavailability of chemical substances and consequently their toxicity for soil organism (Kotschik et al. 2017; Liu et al. 2012; Römbke et al. 2007a). In the current ERA of chemicals, differences in soil characteristics are neither considered in laboratory studies conducted with only OECD artificial soil, nor in field studies performed usually on one site with only one soil type. If differences in soil characteristics are not considered, this can lead to a bias in the results (Römbke et al. 2007a).

In studies examining the toxicity of chemicals on the earthworm species *E. fetida* or *E*. *andrei* in natural soils, the toxicity of chemicals can vary statistically significantly between different soils (Kotschik et al. 2017; Liu et al. 2012). But not only the toxicity of some chemicals can vary between natural soils, also the number of juveniles in the untreated controls can vary greatly between soils (Römbke et al. 2006). Temperature, food quality, pH, and soil moisture have a direct influence on the number of juveniles (Jänsch et al. 2005). The temperature in the tests is standardised 20 ± 2 °C and feeding is done with cattle dung as stated in the OECD guideline 222. And many of the tested natural soils in literature have values near or within the optimum pH 5 to 7 for reproduction of *E. fetida/andrei* (Chelinho et al. 2014; Kotschik et al. 2017; Römbke et al. 2006). However, the optimal moisture in natural soils for reproduction is poorly understood and previous studies on the suitability of natural soils in toxicity testing with *E. fetida/andrei* were performed with a soil moisture recommended for standard studies in OECD artificial soil (Chelinho et al. 2014; Liu et al. 2012, 2013; Garcia et al. 2011; Silvia et al. 2009; Römbke et al. 2006, 2007a). Researchers report the soil moisture as a percentage of maximum water holding capacity (WHC_max_). However, this practical method leads to disadvantages in the comparability between soils: the same percentage value of WHC_max_ may mean different *actual soil water availability*^1^ for earthworms in different soils. A percentage measure cannot not reflect the tension with which water is bound to soils with different porosity.

In this study, the influence of various soil properties on the reproduction toxicity of carbendazim on *E. fetida* was investigated by using natural soils in extended laboratory OECD 222 (2016) earthworm reproduction tests under uniform soil water availability.

We selected carbendazim as a fungicide controlling ascomycetes, fungi imperfecti, and basidiomycetes on a wide variety of crops, including bananas, cereals, cotton, fruits, grapes, mushrooms, sugarbeet, soybeans, tobacco, and vegetables (PubChem 2022). Carbendazim is the toxic reference substance in earthworm field studies (ISO 11268-3 2015) and results in statistically significant reductions of earthworm populations (Ellis et al. 2007). It disrupts the conduction velocity in the giant nerve fibres of earthworms (Drewes et al. 1987) and alters burrowing behaviour (Ellis et al. 2010). Carbendazim is a weak base (pK_a_ 4.48) with low solubility in water and a log P_ow_ of 4.93, which is strongly adsorbed by most soils (Römbke et al. 2020, Ellis et al. 2007). Liu et al. (2013, 2012) found mean lethal effect concentrations of the weak base carbendazim to *E. andrei* in different agricultural soils (LC_50_ 3 to 35 mg a.i kg^−1^ sdw) inversely correlated with soil pH.

Using *E. fetida* does not cover the variability of species specific responses to chemicals (Robinson et al. 2021) and the natural habitat of this “compost worm” are highly organic forest soils and man-made accumulations of organic materials (Jänsch et al. 2005). However, investigating the model organism *E. fetida* recommended in the OECD 222 (2016) guideline in typical agricultural soils is a first step and has the potential to expand the understanding of ecotoxicological studies with soil organisms. The use of natural field soils with various properties provides a more application-oriented approach because it helps to evaluate the risks for the subsequent application of the products more realistically.

Four natural agricultural reference soils (Luvisol, Cambisol, Rendzina and Podsol) from the RefeSol system (Fraunhofer Institute, Germany) were selected that are representative for agricultural use in mid-latitudes (Bussian et al. 2005) and were compared to the OECD artificial soil. The two selected soils classified as Luvisol and Cambisol belong to the worldwide most commonly agricultural used soil types (Amelung et al. 2018).

The first aim of the study was to establish the usage of natural agricultural reference soils with a uniform pF value in the OECD 222 (2016) guideline for an extended earthworm reproduction testing. The second aim was to understand how the toxicity of carbendazim on the reproduction of *E. fetida* is affected by the soil properties of natural soils compared to OECD artificial soil.

## Material and methods

### Test organism

Earthworms of the species *E. fetida* were taken from a synchronised culture at Eurofins Agroscience Services in Niefern-Öschelbronn, Germany. The worm cultures were maintained at 21 ± 1 °C in aerated plastic boxes with moist bark compost mixture and fed with ground oatmeal. The day before starting the test, the worms were acclimatised in untreated moist soil of the subsequent test. The selected adult worms were between seven and eight months old, each worm with a distinct clitellum and a weight between 300 and 600 mg.

### Test soils

The four soils 02-A, 03-G, 04-A and 06-A of the soil reference system RefeSol originated from natural sites were supplied by the Fraunhofer Institute in Schmallenberg, Germany. The letters indicate that the soils were formerly used as arable land (A) or grassland (G), respectively. The soils were air-died and sieved (<2 mm). The OECD standard artificial soil was prepared at the laboratory of Eurofins Agroscience Services Ecotox GmbH in Niefern-Öschelbronn, Germany and consisted of 69.6% quartz sand, 20% kaolin clay, 10% air dried and sieved (<4 mm) peat, and 0.42% calcium carbonate.

### Soil properties

The grain size was determined using a combined method according to DIN ISO 11277 (2002) with wet sieving of fractions 0.063–2 mm and pipette analysis of fractions <0.063 mm with two replicates. The texture class was classified according to WRB (2014) and pH values were determined according to VDLUFA (2012) using two replicates. Total carbon content (TC) and total organic carbon content (TOC) were determined according to DIN ISO 15936 (2012) with two replicates, respectively. Organic matter (OM) was calculated by multiplying TOC by the factor 1.72 (Blume et al. 2011). Cation exchange capacity (CEC) was determined according to DIN ISO 13536 (1997) with buffered barium chloride solution and detected with ion chromatography using two replicates. Microbial biomass was determined with substrate induced respiration according to DIN ISO 14240-1 (1997) and WHC_max_ was determined in accordance with DIN ISO 11268 (2015) with three replicates, respectively. Soil water contents and dry substance were determined according to Blume et al. (2011) with three replicates, respectively. Soil dry density was determined according to (Blake and Hartge 1986) using three replicates.

### pF values

For interpolations of pF values, the soil water matric retention curves (pF curves: the relation between volumetric water content and soil water matric potential), were determined for all soils. First, several pF values were measured in accordance with DIN EN ISO 11274 (2020) for all soils using three replicates. Secondly, interpolations between the measured values for the soil water retention curves were done with the formula of the van Genuchten model (van Genuchten 1980) for RefeSol 02-A, 03-G, 04-A and OECD substrate. For RefeSol 06-A a better fitting was achieved with the formula of Durner bi-modal pore model (Durner 1994). For estimation of the parameters in the formulas the add-in *Solver* in *Microsoft Excel* (version 2110) was used. For this purpose, the *root-mean-square error* of the measured volumetric water contents versus the estimated volumetric water contents were fitted to a minimum.

Gravimetric water contents corresponding to the volumetric water content of pF values were calculated by multiplying the volumetric water content with the density of water and dividing by soil dry density.

### Test substance

The fungicide carbendazim (methyl benzimidazol-2-yl-carbamate) was tested as the formulation Carbomax 500 Sc (nominal 500 g l^−1^, Sumitomo Chemical, Brazil). The analysed content of carbendazim was 492 g L^−1^ in the formulation (EAG 2021). Aqueous solutions were mixed into the soils. Each concentration was set up from a prepared stock solution.

### Earthworm reproduction toxicity test

The effect of carbendazim on the reproduction of *E. fetida* in the four natural reference soils and in OECD artificial soil was investigated according to OECD 222 (2016). The controls for each soil were tested with eight replicates. Each of the five soils was tested with eight concentrations of carbendazim, each concentration with four replicates. Test concentrations of carbendazim were based on the results of range-finding tests. RefeSol 02-A was tested with 0.030, 0.051, 0.086, 0.146, 0.247, 0.418, 0.708 and 1.20 mg a.i. kg^−1^ sdw, RefeSol 03-G and 04-A were tested with 0.200, 0.301, 0.453, 0.682, 1.03, 1.55, 2.33 and 3.50 mg a.i. kg^−1^ sdw. RefeSol 06-A was tested with 0.150, 0.217, 0.314, 0.455, 0.659, 0.954, 1.38 and 2.00 mg a.i. kg^−1^ sdw. OECD artificial soil was tested with 0.200, 0.395, 0.593, 0.889, 1.33, 2.00, 3.00 and 4.50 mg a.i. kg^−1^ sdw.

For each soil and concentration, aqueous carbendazim solutions were mixed homogeneously into the soils with a spiral stirrer attached to a drilling machine for three minutes. An amount of 500 g (sdw) of the prior treated soil was filled into test vessels (white polypropylene trays, 160 × 110 × 65 mm with a transparent lid with holes for air exchange). For pre-moistened OECD artificial soil, the final water content was achieved with application of the carbendazim solution. To prevent soil compression, for all natural soils the application was done on air-dried to slightly moist soil and the final moistening was done directly after application for each replicate separately. Samples for determination of soil water content and pH values of the test start were taken from separate replicates without worms on day two of the test, when water equilibrium was assumed.

Ten adult worms per replicate were placed on the soil surface directly after application and final moistening. Cow dung was added weekly as food supply during the first 28 days. After 28 days of exposure the adult worms were removed, counted, rinsed with water, dried with paper towels and weighted. On day 56 the hatched juveniles were extracted with a 55 °C water bath, collected from the soil surface and counted.

### Toxicity exposure ratio

In the current ERA of soil organisms in the EU, the toxicity exposure ratio (TER) is calculated by dividing the toxic endpoint of laboratory experiments (no observed effect concentration (NOEC) or EC_10_) by a predicted environmental concentration in the soil (PEC_soil_) (SANCO 2002; OECD 2001). The quotient of TER is then compared to a trigger value (also called assessment factor or safety factor). The trigger value accounts for intra- and inter-laboratory variation, differences in sensitivity among species and other uncertainties such as differences between agricultural field soils that are not tested (Chapman et al. 1998). If the TER is higher than the trigger value, no risk is expected for the respective organisms. If it is lower, higher-tier studies must be carried out as further testing requirements in the approval procedure of PPP to investigate the risk under field conditions. For the reproduction toxicity of *E. fetida* this trigger value is 5 (Sanco 2002). Moreover, when log K_ow_ is >2.0, which is the case for the substance carbendazim, the endpoints generated in OECD artificial soil are corrected by dividing by a factor of 2 before calculations of TER (EFSA 2010; SANCO 2002). For the natural soils tested in this study this correction was not undertaken.

### Statistical data analysis

The effect concentrations at which 10% and 50% reduction in the number of juveniles on day 56 compared to the control occurred (EC_10_ and EC_50_) were calculated with the statistic software *ToxRat* (version 3.3.0). For EC_10_ and EC_50_ calculation, the models that fit the best were selected according to *r*^*2*^ and *Akaike Information Criterion* and were a point estimation from non-linear 3-parametric logistic regressions. For all regression models it was confirmed that a statistically significant variance is explained by the regression model with a *one-way anova* and that there is no statistically significant lack of fit (F-test: *p* > 0.05). Moreover, statistical comparisons among the EC_10_ and EC_50_ were conducted using the *software R* (version 4.0.0.). The comped function (*comped*) in the *drc* package (Ritz et al. 2015) developed from a ratio-based method was used for pairwise comparisons (Wheeler et al. 2006).

To investigate which soil parameters in the examined set of soils and substrate are related to the toxicity of carbendazim, exploratory analyses were performed: Principal component analyses (PCA) were performed with the statistical software *CANOCO 5* (version 5.12) to quantify the relative importance of the soil parameters in explaining the EC_10_ and EC_50_ values of carbendazim and to find underlying correlations.

## Results

### Soil properties

The natural reference soils are classified as various soil types and therefore show large differences in their characteristics (Table 1). The textures of these soils ranged from sand to clayey silty loam, TOC ranged from 1.03 to 4.48% and OECD artificial soil with 10% peat is within this range. Moreover, the five soils show a wide range of pH, TOC, OM, CEC, microbial biomass, WHC_max_ and gravimetric saturation water content (θ_s grav_). The four natural soils show a wide range in the nutrients contents of N, P, K and Mg, as well.

**Table 1:** Characteristics of the selected natural RefeSol soils and the OECD artificial soil with 10% peat (mean values ± standard deviation)

Soil	Soil classification	Soil texture class	Sand	Silt	Clay	pH	pH	TC	TOC
	WRB	WRB	DIN [%]	DIN [%]	DIN [%]	0.1 M CaCl_2_	H_2_O	[% C]	[% C]
RefeSol 02-A	Stagnic Luvisol^a^	Si (silt)	4.0 ± 0.1	89.5 ± 0.2	6.5 ± 0.3	6.21 ± 0.04	6.78 ± 0.25	1.05 ± 0.00	1.03 ± 0.00
RefeSol 03-G	Eutric Cambisol^a^	SiL (silt loam)	21.8 ± 0.5	66.6 ± 0.6	11.6 ± 0.1	5.62 ± 0.06	6.42 ± 0.06	4.48 ± 0.01	4.48 ± 0.01
RefeSol 04-A	Gleyic Podsol^a^	LS (loamy sand)	81.5 ± 0.7	15.5 ± 0.6	3.0 ± 0.1	5.32 ± 0.02	6.22 ± 0.09	3.14 ± 0.02	3.14 ± 0.02
RefeSol 06-A	Cambic Rendzina^a^	SiCL (silty clay loam)	11.8 ± 0.1	53.7 ± 0.0	34.5 ± 0.1	7.21 ± 0.04	7.54 ± 0.14	2.82 ± 0.00	2.72 ± 0.02
OECD 10%	-	SL (sandy loam)	76.1 ± 0.3	15.0 ± 0.7	8.8 ± 0.4	5.61 ± 0.06	5.96 ± 0.07	4.11 ± 0.07	4.11 ± 0.07

Soil	Total N	TOC/N	OM	CEC	P_CAL_	K_CAL_	Mg_CaCl2_	Biomass	WHC_max_	θ_S grav_	*ρ*_*s*_
	[g kg^−1^]		[%]	[mmol kg^−1^]	[mg kg^−1^]	[mg kg^−1^]	[mg kg^−1^]	[mg kg^−1^ C_mic_]	[%]	[%]	[g cm^−3^]
RefeSol 02-A	1.14^a^	9.0	1.78	114 ± 1	45^a^	92^a^	37.8^a^	794^a^	46.6 ± 1.1	41.0 ± 2.2	1.19 ± 0.04
RefeSol 03-G	4.9^a^	9.1	7.72	172 ± 2	172^a^	357^a^	141.5^a^	1711^a^	65.4 ± 0.1	58.0 ± 2.8	0.93 ± 0.03
RefeSol 04-A	1.52^a^	20.7	5.41	164 ± 3	61^a^	92^a^	53.5^a^	332^a^	33.0 ± 0.4	23.4 ± 1.1	1.45 ± 0.02
RefeSol 06-A	3.02^a^	9.0	4.69	108 ± 3	86^a^	380^a^	44.6^a^	918^a^	53.7 ± 0.8	57.5 ± 1.0	0.98 ± 0.03
OECD 10%	–	–	7.09	99 ± 1	–	–	–	194 ± 1.4	75.9 ± 0.5	63.5 ± 0.3	0.91 ± 0.02

Given are world reference base (WRB) soil classification, texture class (WRB), texture (DIN), values of total carbon (TC), total organic carbon (TOC), total nitrogen (N), organic matter (OM), cation exchange capacity (CEC), phosphorus (P), kalium (K), magnesium (Mg), microbial biomass (C_mic_), maximum water holding capacity (WHC_max_), gravimetric saturation water content (θs_grav_), and soil dry density (*ρ*_*s*_)

^a^Data provided by Fraunhofer Institute Schmallenberg, Germany: Total N, P/K-_CAL_ and Mg_CaCl2_ according to VDLUFA (2012), C_mic_ according to DIN ISO 14240-2 (2011)

**Fig. 1:**
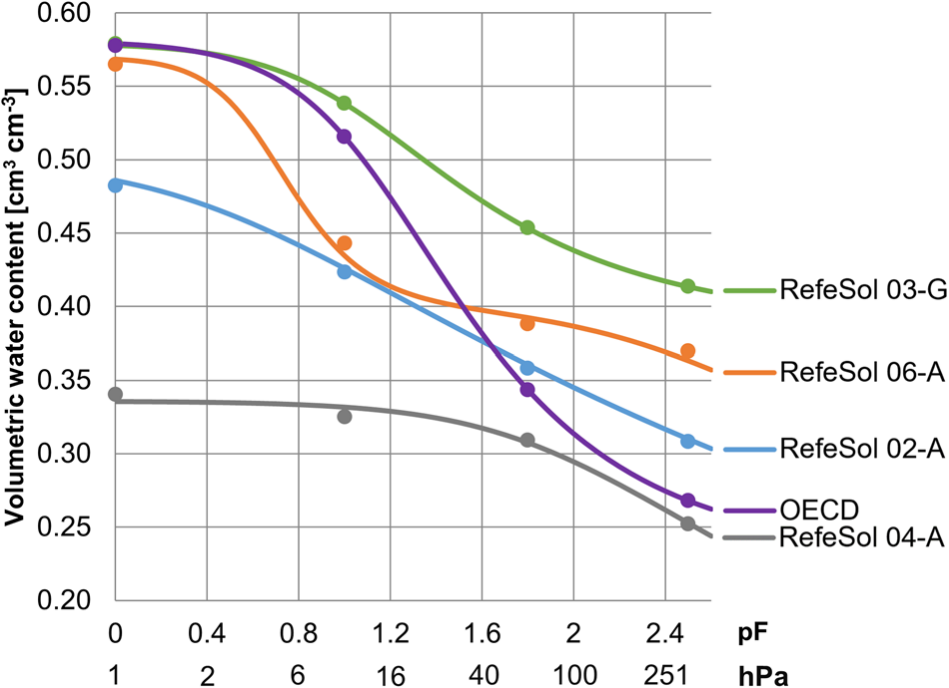
Soil water pF curves of four examined natural soils RefeSol 02-A, 03-G, 04-A, 06-A and OECD artificial soil based on measurements (means shown as dots) and van Genuchten (1980) function or bimodal pore model according to Durner (1994) covering volumetric water content values from saturation water content (pF 0) to the upper range of field capacity (pF 1.8/2.5). Values in hPa are rounded

A model fitting of the soil water pF curves was achieved with *root-mean-square error*s smaller one that were 1.64E-7, 4.97E-7, 1.96E-7, 4.20E-3 and 0.146 for RefeSol 02-A, 03-G, 04-A, 06-A and OECD artificial soil, respectively. Regarding pF values and the water retention in the upper range of field capacity (Fig. 1), the soils show large differences which corresponds to the differences in texture and organic carbon content between the examined soils. The peaty and sandy OECD artificial soil for example, has a high volumetric water content at low pF values 0 to 0.5, comparable to the highly organic and loamy RefeSol 03-G, managed as grassland. With rising pF values there is a steep slope of the curve of OECD artificial soil and from pF 2 it resembles more the sandy RefeSol 04-A with comparable lower capacity for saving water.

The soil moisture for the earthworm reproduction tests was set to the uniform value of pF 1.6 in all soils (Table 2). The pF 1.6 corresponds to 55% of WHC_max_ in the OECD artificial soil which is according to 40 to 60% of WHC_max_ recommended in OECD 222 (2016). With this, the corresponding percentage utilisation of WHC_max_ to pF 1.6 of the four natural soils ranged between 66.2 and 77.4%.

**Table 2:** Different measures for soil moisture in the earthworm reproduction toxicity test corresponding to pF 1.6/θ_vol_ that was based on the soil water pF curves in Fig. 1 and calculated with soil dry density and WHC_max_ from Table 1

Soil	pF	θ_vol_ [cm^3^ cm^−3^]	θ_grav_ [%]	% of WHC_max_
RefeSol 02-A		0.374	31.6	67.8
RefeSol 03-G		0.472	50.6	77.4
RefeSol 04-A	1.6	0.317	21.8	66.2
RefeSol 06-A		0.397	40.4	75.3
OECD 10%		0.381	41.8	55.0

*θ*_*vol*_ volumetric soil water content, *θ*_*grav*_ gravimetric soil water content, *% of WHC*_*max*_ percentage utilisation of WHC_max_

### Validity and performance

For all soils, the validity criteria as described by OECD 222 (2016) were fulfilled. Adult mortality in the controls over the initial four weeks did not exceed 10%, mean number of juveniles in the controls was ≥30 and the coefficient of variation for number of juveniles in controls were below 30% (Table 3).

The test conditions were kept constant during the test. Soil water content for all treatments did not vary more than 10% from the target water content and from that at the test start.

**Table 3:** Control performance in the earthworm reproduction toxicity tests

Soil	Mean control adult mortality [%]	Mean control number of juveniles ± SD	Control CV [%]
RefeSol 02-A	0.0	244 ± 29	12.0
RefeSol 03-G	0.0	231 ± 33	14.2
RefeSol 04-A	2.5	231 ± 42	18.2
RefeSol 06-A	0.0	323 ± 58	17.9
OECD 10%	0.0	277 ± 32	11.5

*SD* standard deviation, *CV* coefficient of variation

### Reproduction toxicity

**Fig. 2:**
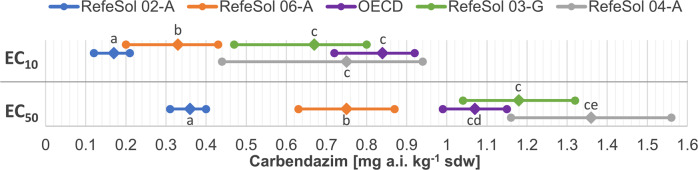
Spectrum of the EC_10_ and EC_50_ values for *E. fetida* reproduction in the four examined natural soils RefeSol 02-A, 03-G, 04-A, 06-A and OECD artificial soil. EC_10_ and EC_50_ values are shown as diamonds, upper and lower 95% confidence limits are shown as dots. EC_10_ or EC_50_ values marked with the different letters are statistically significantly different from each other (*p* ≤ 0.05)

The number of juveniles of *E. fetida* on day 56 in each soil were affected in a concentration related manner (Fig. S1, Supplementary Information). The EC_10_ and EC_50_ values for reproduction differed among the five soils and a cluster of three of the soils is visible (Fig. 2). Highest toxicity was found in RefeSol 02-A, intermediate toxicity in RefeSol 06-A and lowest toxicity in RefeSol 03-G, 04-A and OECD artificial soil, respectively. In RefeSol 02-A, EC_10_ values for reproduction of *E. fetida* were 1.9-times lower than in RefeSol 06-A, 3.9-times lower than in RefeSol 03-G, 4.4-times lower than in RefeSol 04-A and 4.9-times lower than in OECD artificial soil. Moreover, in RefeSol 02-A, EC_50_ values were 2.1-times lower than in RefeSol 06-A, 3.0-times lower than in OECD artificial soil, 3.3-times lower than in RefeSol 03-G and 3.8-times lower than in RefeSol 04-A (Table 4).

**Table 4:** EC_10_ and EC_50_ values with 95% confidence limits in the earthworm reproduction toxicity tests

Soil	EC_10_	EC_50_
RefeSol 02-A	0.17 (0.12–0.21)	0.36 (0.31–0.40)
RefeSol 03-G	0.67 (0.47–0.80)	1.18 (1.04–1.32)
RefeSol 04-A	0.75 (0.44–0.94)	1.36 (1.16–1.56)
RefeSol 06-A	0.33 (0.20–0.43)	0.75 (0.63–0.87)
OECD 10%	0.84 (0.72–0.92)	1.07 (0.99–1.15)

### Toxicity determining soil properties

**Fig. 3:**
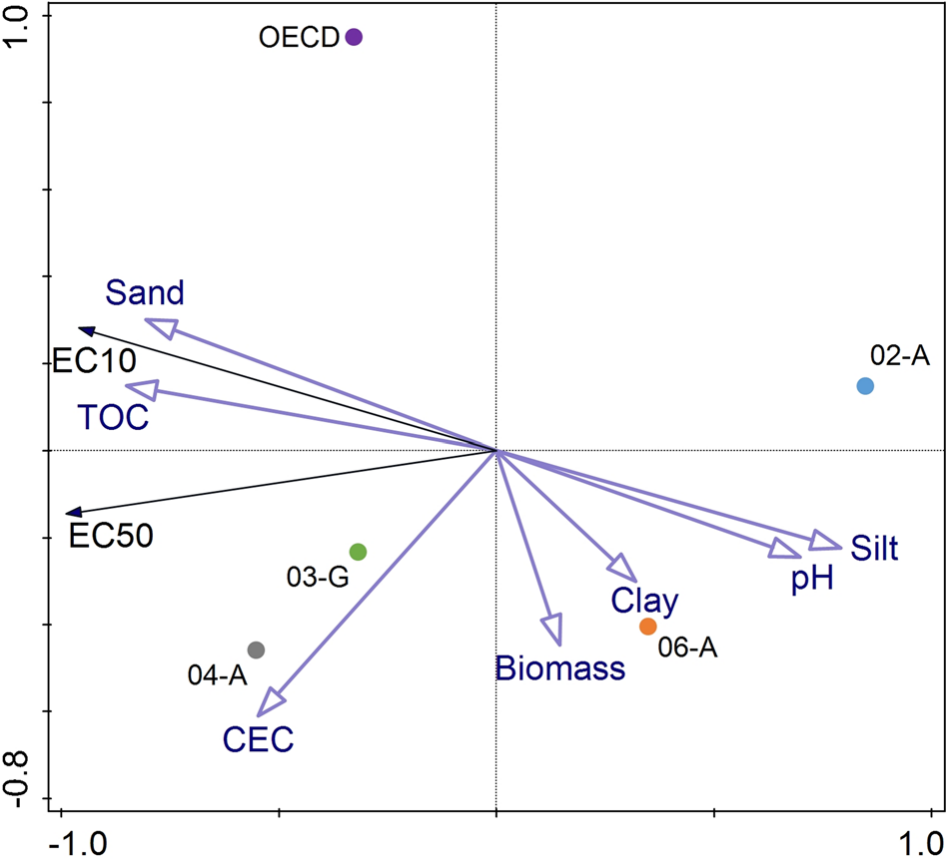
PCA triplot based on EC_10_ and EC_50_ values and seven soil parameters (vectors) of the four investigated RefeSol soils and the OECD artificial soil, as well the soils (coloured dots). Axis 1 (horizontal): Eigenvalue 0.9586, axis 2 (vertical): Eigenvalue: 0.0414

A trend of positive correlation of TOC and sand with EC_10_/EC_50_ and negative correlation of pH and silt with EC_10_/EC_50_ can be seen in the PCA triplot (Fig. 3). The vectors of the soil parameters sand and TOC point in the same direction and have small angles with EC_10_ and EC_50_, hence they show positive correlation with each other. Silt and pH point in the opposite direction and are negatively correlated with EC_10_ and EC_50_. Moreover, the horizontal first axis (first principal component) has an eigenvalue of 0.9586, thus explains 95.86% of the variation. The other soil parameters CEC, biomass and clay are rather short or orthogonal to EC_10_ and EC_50_, additionally the vertical axis in which direction the vectors CEC, biomass and clay mostly run explains only 4.14% of the variation hence they are less related with EC_10_ and EC_50_.

The spatial location of the coloured dots that reflect the soils in the triplot (Fig. 3) shows in relation to the other soils, which soil property they have high or low values of and if they have high or low EC_10_ and EC_50_ values. For example, RefeSol 02-A and RefeSol 06-A both with low EC_10_ and EC_50_ values are on the opposite side of the EC_10_ and EC_50_ vectors in the coordinate system. Another example is RefeSol 06-A that has a high clay content and is close to the clay vector in the coordinate system.

A second PCA was done with natural soils and adding the soil parameters nutrients P, K, Mg, N and C/N (Fig. S2, Supplementary Information). In this PCA, no correlations of nutrients with EC_10_ and EC_50_ of earthworm reproduction to carbendazim in the natural soils were found.

### Toxicity exposure ratio

In accordance with the current lower tier procedure of risk assessment in the European Union, TER values for carbendazim were calculated for four representative crops using EC_10_ values of the present study and literature PEC_soil_ values. They were compared to TER values of a EFSA peer review of risk assessment of carbendazim (EFSA 2010).

TER values were between 1.8 and 22.7, and therefore below and above the trigger value of 5, depending on the soil type and crop species (Table 5). For RefeSol 03-G and 04-A, none of the TER values was below 5. For RefeSol 06-A and the OECD artificial soil, the TER was below 5 for oilseed rape, the crop with the highest PEC_soil_. For RefeSol 02-A, where the highest toxicity was observed, the TER was below 5 for three out of four crops.

**Table 5:** TER values using NOEC or EC_10_ and PEC_soil_ values from a EFSA peer review of risk assessment of carbendazim (EFSA 2010)

PEC_soil_ of crops^a^ [mg a.i. kg^−1^ sdw]		TER: $\frac{{{{{\rm{NOEC}}}}/2}}{{{{{\rm{PEC}}}}}}$	TER: $\frac{{{{{\rm{EC}}}}_{10}/2}}{{{{{\rm{PEC}}}}}}$	TER: $\frac{{{{{\rm{EC}}}}_{10}}}{{{{{\rm{PEC}}}}}}$			
		EFSA OECD^a^	OECD	RefeSol			
				02-A	03-G	04-A	06-A
Cereals	0.049	12^a^	8.6	3.5	13.7	15.3	6.7
Maize	0.061	10^a^	6.9	2.8	11.0	12.3	5.4
Sugar beet	0.033	18^a^	12.7	5.2	20.3	22.7	10.0
Oilseed rape	0.093	7^a^	4.5	1.8	7.2	8.1	3.5

^a^Data according to EFSA (2010)

## Discussion

This study shows that a successful, valid and reproducible usage of natural agricultural soils in the OECD 222 (2016) earthworm reproduction test scheme is possible with the use of the measure pF for soil moisture adjustment that addresses the variability in water-retaining of natural soils. There were clear differences up to 4.9-fold in the toxicity of carbendazim on the reproduction of *E. fetida* between the examined natural soils that were attributable to a combination of the two soil parameters TOC and pH. The differences in the toxicity between the natural soils leads to TER values above or below the safety factor, subsequently resulting in different outcomes of the ERA.

### Soil water

The utilisation of the pF value 1.6 (−40 hPa) to adjust the soil moisture provided a comparable and sufficient soil water availability and resulted in a reproduction performance of *E. fetida* at the same level as OECD artificial soil with mean number of juveniles ranging between 231 and 323. With this uniform pF, the percentage of WHC_max_ was higher than 60% for all natural soils. Using the range of 40 to 60% of WHC_max_ as stated in OECD guideline 222 provides a simple method for OECD artificial soil, but it can neither provide a comparable soil water availability between natural soils, nor suitable moisture conditions for earthworm reproduction in fine textured soils.

For fine textured silty clay loam RefeSol 06-A, a 60% usage of WHC_max_ would correspond to pF 3.2 (−1585 hPa and 32% θ_grav_) and a low soil water availability for earthworms. No literature data regarding pF values was found for *E. fetida or E. andrei*. For the earthworm species *Aporrectodea caliginosa*, cocoon production is already negatively affected by pF values larger than pF 2.1 (−120 hPa) (Holmstrup 2001). For *Allolobophora chlorotica* cocoon production is totally inhibited at pF 3 (−1000 hPa) (Evans and Guild 1948). Using 40 to 60% of WHC_max_ in ecotoxicological laboratory studies with natural soils can therefore lead to a bias in the earthworm reproduction performance and comparisons between soils or studies are subject to uncertainties or even invalid studies.

In addition, the fate and behaviour of substances in soils is influenced by the soil moisture regime (Beulke et al. 2004; Willkommen et al. 2021). From equilibrium partitioning theory, the chemical potential of a substance in soil is assumed to be equivalent to the concentration of the chemical in pore water (Ronday et al. 1997). Many studies show that substances must be in a dissolved form to be bioavailable to earthworms and that the concentration of a substance in the pore water is linked to the toxicity for both oral and dermal uptake route (Vijver et al. 2003). With increasing soil water, the exchange surfaces increase and the diffusion of PPP to these surfaces is facilitated (Roy et al. 2000). This means, depending on the pF value, a substance can be present in a different ratio dissolved in soil pore water and adsorbed to the soil matrix, which leads to a different toxic potential. Therefore, when investigating the toxicity between natural soils in laboratory studies, beside soil sorption parameters also the soil water should be taken into account. A research question that could be addressed in future is, to which extend environmental stress on soil organisms like drought and flooding events have an influence on the toxic effect of substances.

### Toxicity and soil parameters

There were clear differences in the toxicity of carbendazim on the reproduction of *E. fetida* between the examined natural soils that can be explained with the soil parameters TOC and pH.

In the PCA, TOC and sand tended to be positively correlated with the EC_10_ and EC_50_ values and pH and silt tended to be negatively correlated with the EC_10_ and EC_50_ values. Oppositely, the soil parameters CEC, clay, microbial biomass and the plant nutrients N, P, K and Mg did not show relations with the toxic endpoints in the PCAs.

### TOC and pH

Statistically significantly different EC_10_ with a factor up to 4.9 and EC_50_ with a factor up to 3.7 between the five soils of the present study were found (EC_50_ 0.36 to 1.36 mg a.i. kg^−1^ sdw). This range in EC_50_ is comparable to the range of values found in literature data with OECD artificial soil (10% peat). Silva et al. (2009) reported low values (EC_50_ 0.39 mg a.i. kg^−1^ sdw), Chelinho et al. (2014) reported values in the mid-range (EC_50_ 0.89 mg a.i. kg^−1^ sdw) that are comparable to values in artificial soil of the present study (EC_10_ 0.84, EC_50_ 1.07 mg a.i. kg^−1^ sdw) and Sousa et al. (2017) found higher values (EC_10_ 1.5, EC_50_ 2.3 mg a.i. kg^−1^ sdw). This range of variation in the values could explained by the differences between variable sources of artificial soil components. Especially peat can limit the comparability of OECD substrates in different laboratories because peat of different origins can lead to organic carbon contents in the OECD substrate varying from 1.4 to 6.0% (Hofman et al. 2009). Sousa et al. (2017) compared the toxicity of carbendazim to earthworm reproduction in OECD substrate with 10% peat (TOC 4.4%; EC_10_ 1.5, EC_50_ 2.3 mg a.i. kg^−1^ sdw) and 5% peat (TOC 1.9%; EC_10_ 0.8, EC_50_ 1.6 mg a.i. kg^−1^ sdw) of same origin and found statistically significant differences in the reproduction toxicity of *E. andrei*. This influence of TOC is in accordance with the results determined with natural soils and artificial soil in the present study with a TOC ranging from 1.03 to 4.48%. The higher TOC, the less toxic is carbendazim for the reproduction of *E. fetida*.

In contrast, Chelinho et al. (2014) found EC_50_ values of carbendazim in five natural soils varying with a smaller factor of 1.7 (EC_50_ 0.73 to 1.27 mg a.i. kg^−1^ sdw), but with no obvious relationship to TOC and Sousa et al. (2017) found no variations between three natural soils (EC_50_ 0.8 mg a.i. kg^−1^ sdw). Compared to literature data of EC_50_ values determined in natural soils, considerably larger differences were found between the evaluated soils in the present study, which is most likely due to differences in the soil characteristics and the soil water availability as discussed above.

TOC seems not to be the only toxicity determining factor. A causal positive correlation of TOC together with a negative correlation of pH with the sorption of carbendazim to the soil and hence, the EC_10_ and EC_50_ values of earthworm reproduction are in accordance with recent studies regarding sorption and toxicity (Berglöf et al. 2002; Paszko 2006; Ellis et al. 2007; Liu et al. 2012, 2013). Theoretically, the maximum adsorption of carbendazim to soils would occur at a pH close to the pK_a_ value 4.48 of carbendazim (Berglöf et al. 2002; Ellis et al. 2007). As a weak base it accepts protons at low pH and can then adsorb on negatively charged surfaces such as organic matter or can form mineral complexes (Austin and Briggs 1976). Moreover, carbendazim has a pH dependent solubility in water, at low soil pH it is predominantly solved and may then react with soil matrix (pH 4: 29 mg l^−1^, pH 8: 7 mg l^−1^) (EFSA 2010).

Liu et al. (2013) confirmed a decreasing sorption of carbendazim and thus increasing lethal toxicity with rising pH values through amelioration of an arable soil, which is consistent with the results of the present study and several other studies regarding sorption and toxicity (Paszko 2006; Ellis et al. 2007; Liu et al. 2012). Toxicity decreased with the combination of higher TOC values and lower pH (Liu et al. 2012, 2013). Based on literature and results of the present study, the interpretation is as follows: as soil pH decreases, carbendazim is more soluble in soil water and due to its properties as a weak base it is present protonated with a positive charge, it then reacts with the soil matrix and adsorbs to negatively charged surfaces of organic matter (represented by TOC) and is therefore immobilised and less available to *E. fetida*.

This contrasts with findings of Chelinho et al. (2014) who found no relationships between reproduction toxicity of carbendazim to *E. fetida/andrei* and soil parameters and Sousa et al. (2017) who only found TOC as a factor for different toxicities. However, it is subject to many uncertainties when comparing studies with natural soils. On the one hand it is likely that there are differences in the soil water availability due to the usage of the 40 to 60% WHC_max_ between soils in literature studies influencing earthworm reproduction and bioavailability of substances as discussed above. And on the other hand, the features of the selected soils have an influence whether a connection of the soil sorption properties pH and TOC with toxicity of carbendazim can be reflected in a dataset or not.

### Sand and silt

Sand and silt have negligible sorption potential compared to organic matter or clay, because their surface area per unit mass is substantially smaller (Amelung et al. 2018). Moreover, a relation of sand and silt with sorption or toxicity does not agree with literature data (Berglöf et al. 2002; Paszko 2006; Ellis et al. 2007, Liu et al. 2012, 2013). Therefore, a direct causal relation of sand and silt with toxicity can be excluded. There were inter correlations of sand and silt with the explanatory variables TOC and pH. Because these inter correlations are not the case for soils in general, it is likely that they are a spurious relationship due to the small data set. In future research the earthworm reproduction toxicity in a higher number of natural soils could be investigated and as well other pesticides than carbendazim.

### Variable charge, CEC, clay and microbial biomass

Contrasting to the interpretation of an interaction of pH and TOC which influences the toxicity of carbendazim, a substantial sorption of carbendazim could occur even at higher pH values due to variable charged organic matter or clay surfaces that increase in negative charge with increasing pH (Berglöf et al. 2002). The CEC of the investigated soils in the present study showed only low relations to differences in EC_10_ and EC_50_ values. It was determined in buffered barium chloride solution (pH 7 to 8) and hence does not reflect the impact of variable charge. In future research, the effective CEC, determined at the actual soil pH, could be investigated to reveal for variable charged surfaces that could be linked to toxicity of carbendazim or other pesticides.

Despite the high potential sorption capacity of clay due to its huge surface, and opposite to a study (Ellis et al. 2007) there was no correlation between clay and the toxicity of carbendazim in the present study. Moreover, fine textured soils are most prone to bias when WHC_max_ is used as described above.

In the evaluated soils, microbial biomass did not play a role for differences in the observed toxicity to *E. fetida* even though the soils showed large differences in C_mic_. Carbendazim is moderately persistent and showed DT_50_ values of 26 to 40 days under laboratory conditions (EFSA 2010). Additionally, carbendazim and its metabolites are both toxic (Fang et al. 2010; Singh et al. 2016). For these reasons, microbial biomass, and microbial degradation of carbendazim may play a subordinated role in the OECD 222 (2016) test were time for mating and cocoon production is set to 28 days.

### Implication on ERA

Unlike the approach of worst case exposure on inert glass plates in lower tier studies with non-target beneficial organisms (Candolfi et al. 2000), the results of the present study and other studies (e.g. Domene et al. 2012; Amorim et al. 2005) have shown that the use of a standardised artificial soil in lower tier laboratory tests with soil organisms does not reflect a worst case scenario as it ranges among the soils with lowest toxicity. In the present study with carbendazim, integrating an agricultural soil (Luvisol RefeSol 02-A) with a typical low organic carbon content around 1% in the laboratory tests for the exposure assessment has lowered the TER in comparison to OECD artificial soil. For Luvisol RefeSol 02-A, all of the TER were found to be less or equal the assessment factor of 5. This means that the current assessment factor for the evaluation of the TER might have already been “used up” by differences in soil sorption properties like organic carbon content only, without considering for example alteration of effects in the field by co-stressors or without taking into account other earthworm species which have been shown to be more sensitive than *E. fetida/andrei* (Robinson et al. 2021).

Liu et al. (2012) found a ten times higher LC_50_ of carbendazim between two soils with different soil sorption properties. Further, Kotschik et al. (2017) found a three times higher EC_50_ for reproductive toxicity of ethofumesat in a sandy soil compared to a loamy soil. And van Hall et al. (2022) found differences of up to 7-times for EC_50_ values for imidacloprid between two soils.

Christl et al. (2016) compared pairs of studies with no observed effect levels generated in the laboratory with artificial soil soil that failed risk assessment at lower tier (TER < 5) and corresponding no-observed ecologically adverse effect concentrations one year after application generated in earthworm field studies. Their results indicate that the current approach with an assessment factor 5 provides a conservative lower tier risk assessment in comparison to the higher tier field studies. However, no comparison of laboratory studies with TER > 5 and corresponding field results was presented.

A field study of the Federal Environment Agency Germany (Römbke et al. 2020) resembles the findings of the present study in the laboratory since toxicity of carbendazim on natural earthworm populations was in a similar range as that for *E. fetida* in the tested soils. The authors found an EC_50_ of 1089 g carbendazim ha^−1^ for total earthworm abundance one month after application which corresponds to 1.5 mg carbendazim kg sdw^−1^ (calculated according to OECD 216 (2000) assuming a soil dry density of 1.5 g cm^-3^ and a penetration depth of 5 cm). The field soil was a silt loam with pH 7.2 and TOC 1.46 which is plus-minus comparable to soil sorption properties of RefeSol 06-A and 02-A. However, as field studies according to ISO 11268-3 (2015) guideline are performed usually on one site with one soil type only, it remains questionable if the influence of soil properties is adequately covered in the current approach of risk assessment for in soil organisms.

This gap could be closed by using a set of different natural soils in standardised laboratory tests including a soil which is sufficiently representative for use of the pesticide (EFSA 2015). However, the above discussed points suggest, that when using typical agricultural soils in the laboratory at lower tier or as an intermediate tool in the frame of ERA, a suitable assessment factor needs to be developed. Future research could compare the results generated in the laboratory with results from earthworm field studies with the same agricultural soil. Beside differences in soil properties or species specific sensitivity, the exposure scenario and toxicity is for example dependent on climatic conditions like precipitation and drought events (Kaka et al. 2021; Owojori and Reinecke 2010) or changes in temperature (Velki and Ečimović 2015; Römbke et al. 2007b). Therefore, further research both in the laboratory under controlled conditions and under realistic field conditions is required to evaluate the effects of pesticides and the conservatism of the assessment factor under the influence of co-stressors like heat, drought and periodical flooding which are supposed to occur more frequently under human-induced climate change.

## Conclusion

This study shows that a successful, valid and reproducible usage of natural agricultural reference soils (RefeSol) in the OECD 222 (2016) earthworm testing is possible with a high reproduction performance of *E. fetida* at the same level as OECD artificial soil. A pre-requisite for a successful testing is, that comparable soil moisture conditions in natural soils are set with pF values as a measure for soil moisture adjustment that corresponds to the guideline recommendation of WHC_max_ in OECD artificial soil.

Moreover, the substance carbendazim showed different toxicity on the reproduction of *E. fetida* with a factor up to 4.9 between the evaluated soils. This is likely due to an interaction of the soil parameters pH and organic carbon content: an alkaline soil pH together with a low soil organic carbon content leads to a comparable high toxicity of carbendazim. For instance, in case of carbendazim, integrating a typical agricultural soil (Luvisol RefeSol 02-A) in the laboratory tests at lower tier of ERA would have lowered the TER in comparison to OECD artificial soil. This demonstrates, that soil parameters can have a relevant impact on the toxicity of pesticides and is an argument for the supplementation of the present guideline to achieve a substantial improvement of the realism of the test. Exposing the earthworms to pesticides in natural agricultural reference soils with different properties provides a more application-oriented approach and can help in refining the ERA. Instead of a single ecotoxicological endpoint in OECD artificial soil with non-negligible uncertainties, studies with a set of natural soils can provide more realistic results to estimate a spectrum of possible risks of pesticides that may occur in diverse agricultural soils under field conditions. It could serve as a new tool in the ERA and could be used at lower tier or as one component in an intermediate tier based approach.

## Supplementary Information

Supplementary Information

## Footnotes

1 *Actual soil water availability* is determined by the soil water matric potential (often specified as unit pF). It is the tension with which water is bound to the soil and depends to a large extent to the pore sizes and thus the texture of a soil. The smaller a pore, the stronger water is bound. Hence, some fractions of soil water are bound so strongly that they cannot be readily used by the soil biosphere. The matric potential of a soil can be expressed and measured as unit water-tension (hPa/kPa) and is common expressed as logarithmic pF (pF = log |hPa | ). Stahr (2012)
