# Correction to: Natural soils in OECD 222 testing — influence of soil water and soil properties on earthworm reproduction toxicity of carbendazim

**DOI:** 10.1007/s10646-023-02649-4

**Published:** 2023-04-07

**Authors:** Eva Aderjan, Eiko Wagenhoff, Ellen Kandeler, Thomas Moser

**Affiliations:** 1 Eurofins Agroscience Services Ecotox GmbH, Eutinger Straße 24, 75223 Niefern-Öschelbronn, Germany; 2 University of Hohenheim, Institute of Soil Science and Land Evaluation, Emil-Wolff-Str. 27, 70599 Stuttgart, Germany

Correction to: Ecotoxicology (2023)

10.1007/s10646-023-02636-9

In the original publication of the article, the authors noticed the textual errors occurred in the published version. The value for log P_ow_ of carbendazim given in Introduction is incorrect and it should be 1.4–1.5. Consequently, there will be corrections in Material and Methods (section toxicity exposure ratio), in Table 5 and in the description of Table 5 (section results, toxicity exposure ratio), as well. The corrections have no impact on the overall results or the discussion.

In Introduction, seventh paragraph should read “We selected carbendazim as a fungicide controlling ascomycetes, fungi imperfecti, and basidiomycetes on a wide variety of crops, including bananas, cereals, cotton, fruits, grapes, mushrooms, sugarbeet, soybeans, tobacco, and vegetables (PubChem 2022). Carbendazim is the toxic reference substance in earthworm field studies (ISO 11268-3 2015) and results in statistically significant reductions of earthworm populations (Ellis et al. 2007). It disrupts the conduction velocity in the giant nerve fibers of earthworms (Drewes et al. 1987) and alters burrowing behaviour (Ellis et al. 2010). Carbendazim is a weak base (pK_a_ 4.48) with low solubility in water and a log P_ow_ of 1.4–1.5, which is strongly adsorbed by most soils (Römbke et al. 2020, EFSA 2010, Ellis et al. 2007). Liu et al. (2013, 2012) found mean lethal effect concentrations of the weak base carbendazim to *E. andrei* in different agricultural soils (LC_50_ 3 to 35 mg a.i kg^−1^ sdw) inversely correlated with soil pH.”

In section Material and Methods – Toxicity exposure ratio, the paragraph should read “In the current ERA of soil organisms in the EU, the toxicity exposure ratio (TER) is calculated by dividing the toxic endpoint of laboratory experiments (no observed effect concentration (NOEC) or EC_10_) by a predicted environmental concentration in the soil (PEC_soil_) (SANCO 2002, OECD 2001). The quotient of TER is then compared to a trigger value (also called assessment factor or safety factor). The trigger value accounts for intra- and inter-laboratory variation, differences in sensitivity among species and other uncertainties such as differences between agricultural field soils that are not tested (Chapman et al 1998). If the TER is higher than the trigger value, no risk is expected for the respective organisms. If it is lower, higher-tier studies must be carried out as further testing requirements in the approval procedure of PPP to investigate the risk under field conditions. For the reproduction toxicity of *E. fetida* this trigger value is 5 (Sanco 2002). Moreover, when log P_ow_ is >2.0, the endpoints generated in OECD artificial soil are corrected by dividing by a factor of 2 before calculations of TER (EFSA 2010, SANCO 2002). For the natural soils and OECD artificial soil tested in this study this correction was not undertaken.”

In section Results – toxicity exposure ratio, the paragraphs should read **“**In accordance with the current lower tier procedure of risk assessment in the European Union, TER values for carbendazim were calculated for four representative crops using EC_10_ values of the present study and literature PEC_soil_ values. They were compared to TER values of a EFSA peer review of risk assessment of carbendazim (EFSA 2010).

TER values were between 1.8 and 25.5, and therefore below and above the trigger value of 5, depending on the soil type and crop species (Table 5). For OECD artificial soil, RefeSol 03-G and 04-A, none of the TER values was below 5. For RefeSol 06-A, the TER was below 5 for oilseed rape, the crop with the highest PEC_soil_. For RefeSol 02-A, where the highest toxicity was observed, the TER was below 5 for three out of four crops.”

**Table 5:** TER values using NOEC or EC_10_ and PEC_soil_ values from a EFSA peer review of risk assessment of carbendazim (EFSA 2010).

PEC_soil_ of crops^a^ [mg a.i. kg^−1^ sdw]		TER: $\frac{{{{{\boldsymbol{NOEC}}}}}}{{{{{\boldsymbol{PEC}}}}}}$	TER: $\frac{{{{{\boldsymbol{EC}}}}_{10}}}{{{{{\boldsymbol{PEC}}}}}}$	TER: $\frac{{{{{\boldsymbol{EC}}}}_{10}}}{{{{{\boldsymbol{PEC}}}}}}$			
		EFSA OECD^a^	OECD	RefeSol			
				02-A	03-G	04-A	06-A
cereals	0.049	12^a^	17.1	3.5	13.7	15.3	6.7
maize	0.061	10^a^	13.8	2.8	11.0	12.3	5.4
sugar beet	0.033	18^a^	25.5	5.2	20.3	22.7	10.0
oilseed rape	0.093	7^a^	9.0	1.8	7.2	8.1	3.5

^a^Data according to EFSA (2010).

