# Supplementary material for: Natural soils in OECD 222 testing — influence of soil water and soil properties on earthworm reproduction toxicity of carbendazim
Source: Ecotoxicology. 2023 Mar 1;32(4):403–15. doi: 10.1007/s10646-023-02636-9 (PMC10199837; doi:10.1007/s10646-023-02636-9)
Supplement: Supplementary file 1 — Supplementary Information [file 10646_2023_2636_MOESM1_ESM.pdf]

Supplementary Information to:

Natural soils in OECD 222 testing - influence of soil water and soil properties on earthworm reproduction toxicity of carbendazim

Eva Aderjan[1,2*], Eiko Wagenhoff[1], Ellen Kandeler[2], Thomas Moser[1]

[1]Eurofins Agroscience Services Ecotox GmbH, Eutinger Straße 24, 75223 Niefern-Öschelbronn, Germany

[2]University of Hohenheim, Institute of Soil Science and Land Evaluation, Emil-Wolff-Str. 27, 70599 Stuttgart, Germany

*Corresponding author: evaaderjan@eurofins.com

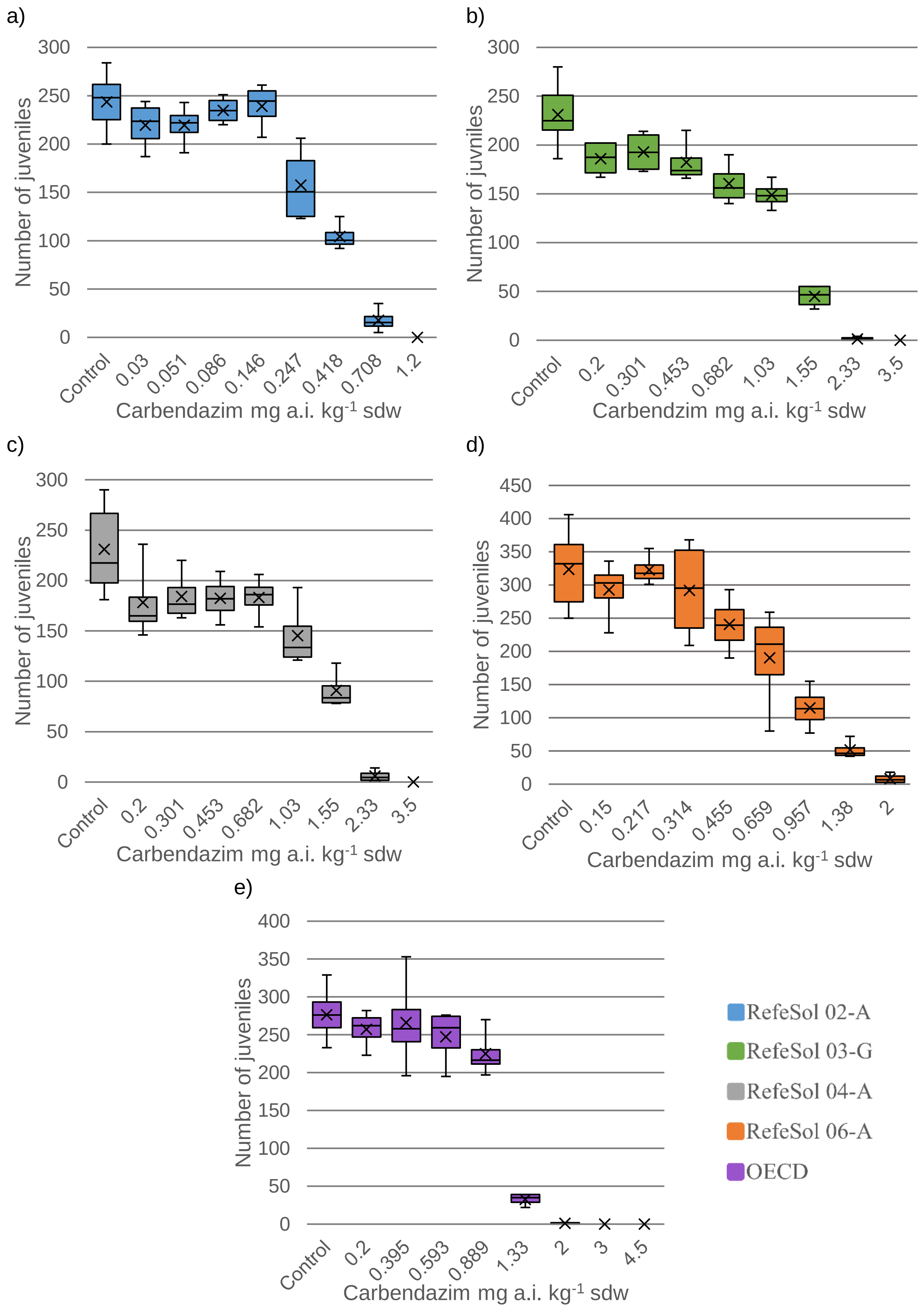

Fig. S1: Boxplots of number of juveniles on day 56 per concentration of RefeSol 02-A (a), RefeSol 03-G (b), RefeSol 04-A (c), RefeSol 06-A (d), and OECD artificial soil (e). Please note the different concentrations in the x-axis and different maximum values of juveniles in the y-axis. Boxes: lower and upper quartiles, whiskers extend to the minimum and maximum values, x sign: mean values, centre line: median.

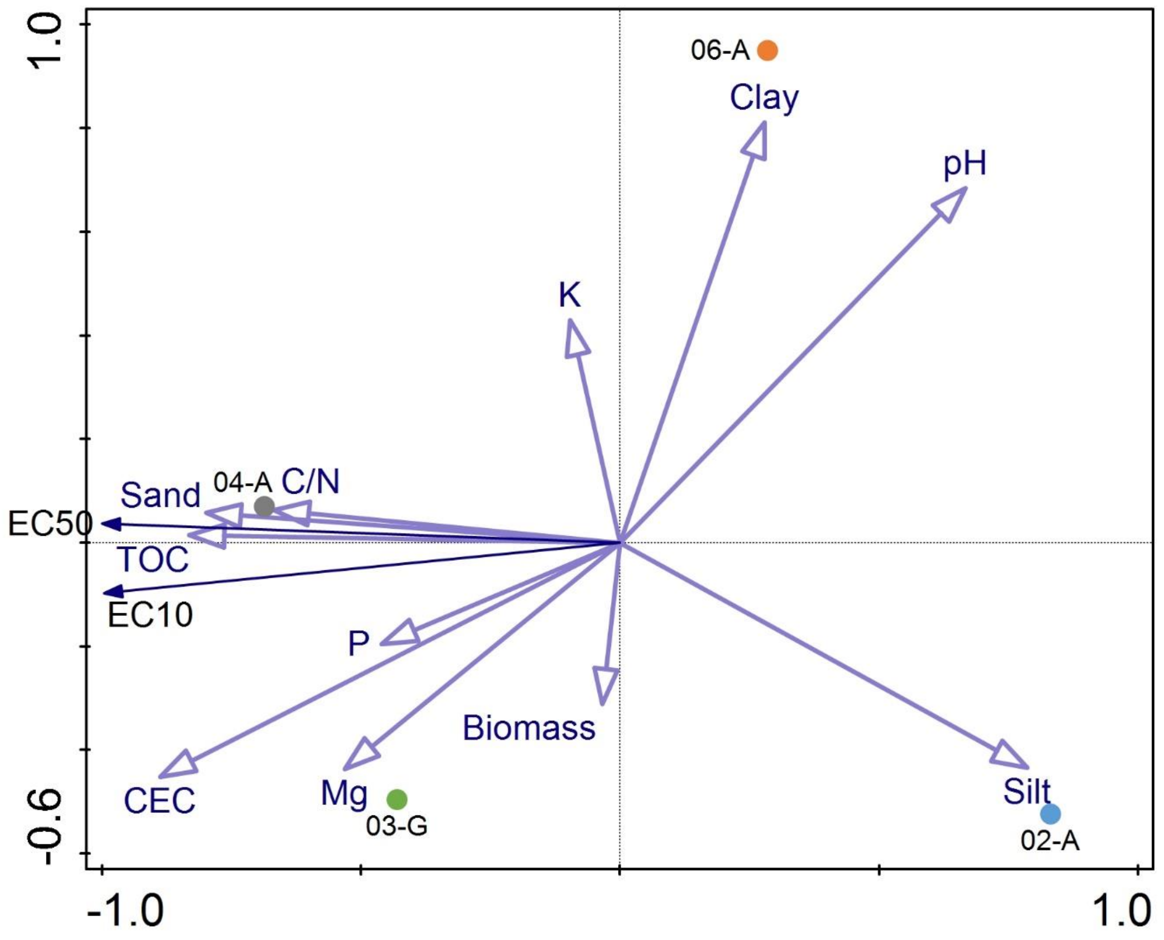

Fig. S2: PCA triplot based on EC_{50} and EC_{10} values and **11 soil parameters** (vectors) of the **four investigated RefeSol soils** (coloured dots). Axis 1 (horizontal): Eigenvalue 0.9965, axis 2 (vertical): Eigenvalue: 0.0035. OECD substrate is not included because of some missing soil parameters
